# Cell culture medium influences RSV G protein glycosylation and recognition by monoclonal antibodies

**DOI:** 10.1186/s12985-026-03318-8

**Published:** 2026-09-29

**Authors:** Nele Engling, Marie-Christin Knittler, Rajarshee Tagore, Martin Ludlow, Albert D.M.E. Osterhaus, Guus F. Rimmelzwaan, Robert Meineke

**Affiliations:** Research Center for Emerging Infections and Zoonoses (RIZ), University of Veterinary Medicine Hannover, Hannover, Germany

**Keywords:** Respiratory syncytial virus, Glycoprotein, Glycosylation, Culture medium, Monoclonal antibody

## Abstract

Respiratory syncytial virus (RSV) glycoprotein G is heavily glycosylated and of variable size following virus growth in transformed cell lines or primary cells. However, it is unknown if the cell culture medium can influence the patterns of glycosylation. Therefore, we tested whether the choice of culture medium can affect the glycosylation of RSV G protein, viral infectivity and antibody recognition. The RSV G ectodomain was produced in HEK293T cells cultured in three different media. Marked differences in molecular weight were observed that were abolished in glycosylation-deficient knockout cells. RSV produced in A549 cells cultured in the same media also differed in relative G protein signals in virus stocks and virus spread. Higher apparent G protein glycosylation was associated with lower binding signals of human F protein-specific monoclonal antibodies to RSV-infected cells. These data show that the cell culture medium can influence biological properties of RSV preparations with consequences for antibody recognition and viral spread.

**Supplementary Information:**

The online version contains supplementary material available at https://doi.org/10.1186/s12985-026-03318-8.

## Main text

Respiratory syncytial virus (RSV) is a leading cause of severe lower respiratory tract infections in infants, young children and older adults [1–3]. Among its surface proteins, the attachment glycoprotein G undergoes extensive post-translational modification, due to 4–5 N-linked and approximately 30–40 O-linked glycosylation sites that increase its apparent molecular weight multiple fold beyond that predicted from its amino acid sequence [4, 5]. These glycans are likely to contribute to antigenic masking and other functional properties of the protein [5–7]. In addition to glycosylation-dependent size heterogeneity, RSV propagated in Vero cells can contain a truncated 55-kDa G protein, compared with the approximately 90-kDa G protein present in HEp-2 cell-grown virus, which is associated with altered infectivity and glycosaminoglycan dependence [8].

The glycosylation profile of G has been reported to be variable. It has been shown that G proteins produced in primary human airway epithelial cultures, including air-liquid interface systems, have an apparent molecular weight of approximately 170 kDa and are more heavily glycosylated than those produced in standard transformed cell lines such as HEp-2 or A549, where G protein species typically range from approximately 55 to 90 kDa [5]. Host cell effects on G protein glycosylation have also been described across continuous cell lines cultured in a single complete medium, where G proteins differed in electrophoretic mobility and in reactivity with G-specific monoclonal antibodies [9]. These differences are functionally relevant, as viruses with the larger glycoforms of the G protein display altered infectivity in standard cell culture systems [5]. Whether such differences are driven primarily by cell identity or by the culture medium used to generate virus stocks has not been studied. In that earlier work the cell line varied while the medium was held constant [9], whereas comparisons between primary and continuous cultures differ in both cell identity and medium [5]. By varying the medium within a single cell background, the present study isolates the contribution of the medium from that of cell identity. Given that culture medium composition can influence the availability of nucleotide sugar donors and the activity of glycosylation pathways [10–13], medium-dependent metabolic effects are a plausible driver of glycosylation heterogeneity.

This issue is also relevant for the recognition of RSV by specific antibody preparations. Approved therapeutic monoclonal antibodies for RSV including Palivizumab, nirsevimab and clesrovimab are directed to the F protein, but virion composition and glycoprotein organization may influence antibody binding and neutralizing activity [4, 7, 14]. Here, we investigated whether the choice of culture medium has an influence on the glycosylation of the RSV G protein and whether this would correlate with viral infectivity and binding of F protein-specific monoclonal antibodies.

**Fig. 1:**
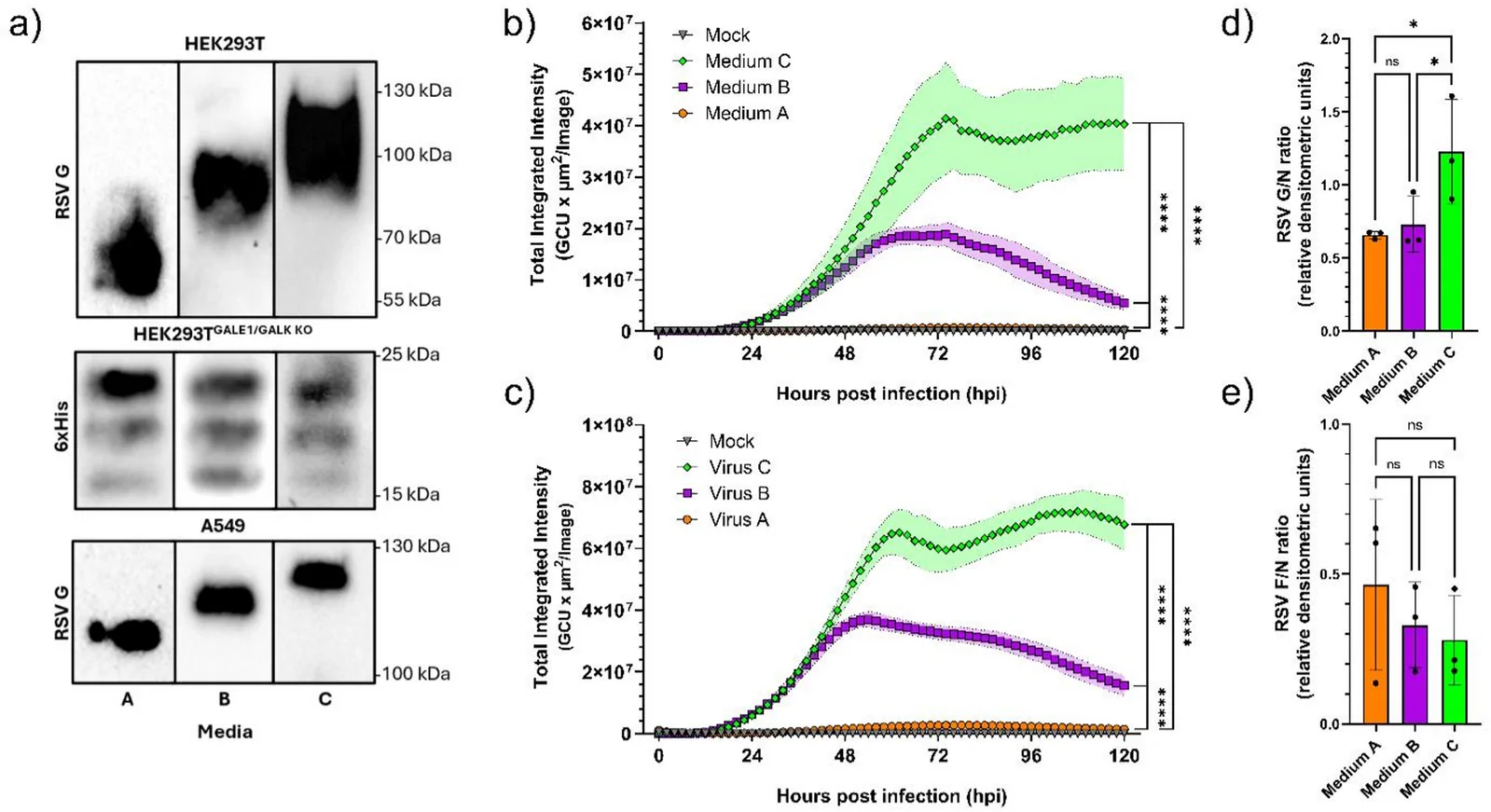
Medium-dependent effects on RSV G protein size, relative protein signals in virus stocks, and viral spread. (**A**) Western blot analysis of soluble G (sG) protein derived from HEK293T cells (top), HEK293T GALE/GALK1 knockout cells (middle), and whole-cell lysates of RSV A-0594-EGFP-infected A549 cells (bottom, 24 hpi), all produced or infected in cell culture medium A, B or C. (**B**) Culture medium-dependent viral spread of RSV A-0594-EGFP in A549 cells infected at an MOI of 1 and cultured in media A, B, or C. GFP integrated fluorescence intensity (GCU × µm²/image; mean ± SD, *n* = 9 from three independent experiments) was quantified over time using an IncuCyte system (Sartorius). Statistical significance was assessed by two-way ANOVA. (**C**) Virus spread following infection with RSV virus stocks A-C in A549 cells infected at an MOI of 1, with medium replaced by Opti-MEM (medium B) after adsorption. GFP integrated fluorescence intensity (GCU × µm²/image; mean ± SD, *n* = 9 from three independent experiments) was quantified over time using an IncuCyte system. (**D**) Relative G protein signal normalised to N protein signal in lysed RSV A-0594-EGFP virus stocks A-C, determined by Western blot densitometry (mean ± SD, *n* = 3). Statistical significance was assessed by one-way ANOVA. (**E**) Relative F protein signal normalised to N protein signal in lysed RSV A-0594-EGFP virus stocks A-C, determined by Western blot densitometry (mean ± SD, *n* = 3). Statistical significance was assessed by one-way ANOVA. *P ≤ 0.05, **P ≤ 0.01, ***P ≤ 0.001, ****P ≤ 0.0001. ns, not significant

To address this, we first used a recombinant protein expression system to test whether the choice of culture medium has an impact on the glycosylation of the RSV G proteins. The soluble form of the RSV protein (sG) was produced in HEK293T cells maintained in medium A, B, or C (see below), and purified proteins were analyzed by SDS-PAGE and Western blot (Fig. 1a; top). The apparent molecular weight (Mw) of sG differed substantially depending on the culture medium used during expression. Protein produced with medium A exhibited the lowest molecular weight (~ 60–90 kDa), that produced with medium B had an intermediate size (~ 100 kDa), and that produced with medium C had the largest Mw (~ 100–130 kDa). To confirm that these differences in Mw were caused by glycosylation, RSV sG was also expressed in HEK293T GALE/GALK1 knockout cells, which are deficient in galactose metabolism [15]. In these glycosylation-deficient cells, the medium-dependent size differences were abolished, and sG was detected as a lower Mw cluster of species (~ 15–25 kDa), consistent with residual heterogeneity from incompletely processed, non-galactosylated glycans that are not eliminated by the GALE/GALK1 knockout (Fig. 1a; mid). To assess whether these medium-dependent glycosylation differences also occur during RSV infection, A549 cells were infected with RSV A-0594-EGFP in media A, B and C and whole-cell lysates were probed for G protein by Western blot 24 hpi (Fig. 1a; bottom). Also, after infection, the same hierarchy in apparent medium-dependent molecular weight was observed as for recombinantly expressed sG protein, although the absolute differences were smaller.

We next assessed whether the choice of cell culture medium has an impact on the infectivity of RSV preparations. To this end, A549 cells were infected with RSV A-0594-EGFP at an MOI of 1 in medium A, B, or C. Because Opti-MEM (medium B) is commonly used for RSV propagation, it served as the reference condition. Virus spread kinetics, quantified by integrated GFP fluorescence intensity over 120 h, demonstrated pronounced medium-dependent differences (Fig. 1b). Virus spread was significantly higher in cultures maintained in medium C than in those maintained in media A and B (*p* ≤ 0.0001).

To determine whether the observed differences in virus spread were dependent on the continued presence of the respective production media, A549 cells were infected with virus stocks produced in media A, B or C at an MOI of 1, based on the infectious titre (TCID50/ml) determined separately for each stock on HEp-2 cells. After viral adsorption, the inoculum was removed and replaced with Opti-MEM (medium B) in all conditions. Differences in virus spread persisted (Fig. 1c). Virus produced in medium C showed the highest spread, virus produced in medium B showed an intermediate phenotype, and virus produced in medium A remained markedly attenuated (*p* ≤ 0.0001). These findings show that the observed differences were not dependent on the continued presence of the production media after adsorption and were associated with the properties of the respective virus-stock preparations, including differences in apparent G protein glycosylation.

We next investigated whether differences in sG glycosylation were associated with relative G protein signals in virus stocks. Virus stocks produced with media A, B or C were adjusted to equal total protein concentrations by Bradford assay, lysed and analysed by Western blot. G protein band intensities were normalised to the corresponding viral N protein signals to calculate relative G/N ratios (Fig. 1d). Because these analyses were performed on clarified, non-purified virus stocks, the G/N and F/N ratios reflect relative protein signals in the stock preparations rather than in purified virions. Virus produced with medium A or B had lower relative G/N ratios than virus produced with medium C (*p* ≤ 0.05 for both comparisons). We also quantified F protein levels in the same virus preparations and normalized the signal to N protein (Fig. 1e). In contrast to the relative G/N ratios, the relative F/N ratios did not differ significantly between viruses produced with media A, B or C (Fig. 1e). As this analysis did not distinguish between pre-F and post-F conformations, it does not exclude medium-dependent differences in F protein conformation.

**Fig. 2:**
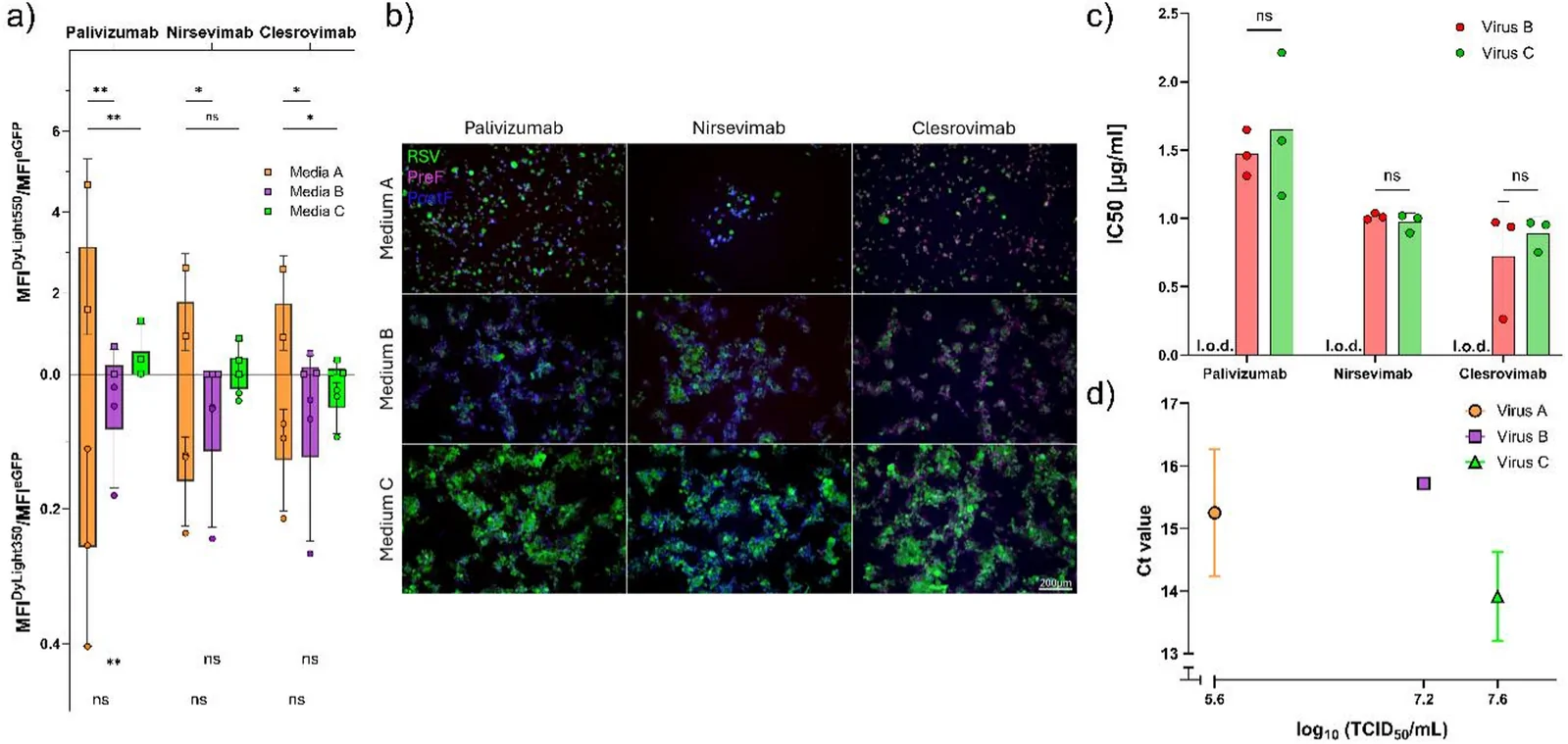
Antibody binding and neutralization of recombinant RSV produced in medium A, B or C. (**A**) Quantification of red (therapeutic F-specific antibodies: palivizumab, which binds both the pre-F and post-F conformations, and the pre-F-specific antibodies nirsevimab and clesrovimab) and blue (post-F-specific control antibody MAB8599) fluorescence intensity, normalized to GFP signal, for infected cells cultured in medium A, B or C (mean ± SD, *n* = 3). Statistical significance was assessed by one-way ANOVA. (**B**) Representative fluorescence images of infected cells stained with palivizumab, nirsevimab and clesrovimab (red) and the post-F control antibody (blue). (**C**) IC50 values of palivizumab, nirsevimab and clesrovimab, against virus produced with medium B or C, determined by 4-PL regression (mean ± SD, *n* = 3 independent experiments). Statistical significance was assessed using fitted IC50 values. (**D**) Scatter plot showing RT-qPCR Cq values versus TCID50 values for virus stocks A-C. *P ≤ 0.05, **P ≤ 0.01, ***P ≤ 0.001, ****P ≤ 0.0001. ns, not significant

Next, we assessed binding of the pre-F-specific antibodies clesrovimab and nirsevimab, and the pre- and post-F-reactive antibody palivizumab, each labelled with a red fluorescent dye, to infected cells. An antibody specific to the post fusion conformation of the F protein was included as control (blue). It served to monitor F protein detection in the same cells independently of the therapeutic antibodies; palivizumab binds both the pre-F and post-F conformations, whereas nirsevimab and clesrovimab are pre-F-specific. Fluorescence signals were normalized to GFP intensity of the RSV A-0594-EGFP reporter virus (Fig. 2a) and corroborated by representative microscopy images (Fig. 2b). The binding signals of palivizumab, clesrovimab and nirsevimab in RSV-infected cells cultured in media B and C were significantly lower than those in cells cultured in medium A. Specifically, binding of palivizumab and clesrovimab was reduced both for medium B and C media (*p* ≤ 0.01 and *p* ≤ 0.05, respectively), and binding of nirsevimab binding was significantly lower for medium B (*p* ≤ 0.05). In contrast, the normalized blue signal representing post-F antibody binding remained largely unchanged for the three media tested. Only a significant reduction of binding was observed between cells cultured in medium A and C in the palivizumab co-staining treatment (*p* ≤ 0.01).

We also determined the neutralising activity of the three F protein-specific antibodies and calculated their IC_50_ values using virus stocks prepared in media B and C. Virus produced in medium A was excluded from this analysis because its attenuated spread kinetics in the IncuCyte-based readout fell below the dynamic range required to reliably resolve a dose-response curve, although its infectious titre was sufficient for assay input. Palivizumab consistently exhibited the highest IC_50_ values (approximately 1.5 µg/ml), whereas nirsevimab and clesrovimab demonstrated greater potency. No statistically significant differences in IC_50_ values were observed between viruses produced in media B and C (Fig. 2c).

To compare the relative RT-qPCR signal and infectious titre of the respective virus stocks, Cq values were plotted against log₁₀ infectious virus titre (TCID50/ml) (Fig. 2d). Virus produced in medium A yielded higher Cq values than virus produced in medium C, whereas its infectious virus titre was approximately 100-fold lower. Thus, the difference in infectious titre was greater than the corresponding difference in relative RT-qPCR signal, consistent with differences in the ratio of infectious to RT-qPCR-detectable viral material between the respective virus-stock preparations.

Collectively, these data show that the choice of culture medium influences the properties of RSV preparations including the extent of glycosylation of the G protein, its relative signal in virus stocks, viral infectivity and binding of F-protein specific antibodies. Because the molecular weight of the RSV G protein differed depending on the medium used with the same cells, and because these differences were abolished using GALE/GALK1 knockout cells, medium composition defined the extent of glycosylation and not cell type specific differences in the glycosylation machinery [10, 11]. This suggests that nutrient and metabolite availability may have contributed to the differences in glycosylation of the G protein previously described between cell lines and primary airway cultures in addition to cell-specific effects [5]. Medium composition is therefore a critical variable when comparing glycosylation patterns between protein expression systems [10–13]. The differential medium-dependent glycosylation patterns of the RSV G protein were also observed after infection of A549 cells, thus not restricted to its overexpression in HEK293T cells. Of interest, the G protein with the highest apparent molecular weight, consistent with more extensive glycosylation, was associated with the highest relative G/N ratio in the virus stocks produced in the corresponding medium C. This association is consistent with a relationship between G protein maturation and relative G protein signal in virus stocks, but does not establish causality. This raises the possibility that post-translational maturation of G contributes to relative G protein signals in virus stocks.

Differences in virus spread persisted when virus stocks produced in media A, B or C were used to infect A549 cells and the inoculum was replaced with standard Opti-MEM after adsorption. This indicates that the observed phenotype was not dependent on the continued presence of the production medium during the subsequent infection period, but does not identify the underlying viral or stock-associated determinant. Equal infectious doses were used for infection, so a difference confined to the initial inoculum would be expected to produce comparable spread curves with a delay rather than a sustained difference in spread. The persistence of the difference is therefore consistent with a stock-intrinsic property that also affects subsequent rounds of infection. The differences in infectious titre between the respective virus preparations were considered together with their relative RT-qPCR signals. Although virus stocks produced in media A and C differed by approximately 100-fold in infectious titre, their Cq values differed comparatively modestly. This observation is consistent with differences in the ratio of infectious to RT-qPCR-detectable viral material between the virus-stock preparations, but does not identify the underlying mechanism. One possible explanation is that the reduced apparent G protein glycosylation of virus produced in medium A could contribute to differences in virus maturation or relative G protein signals in virus stocks, with consequences for infectivity [5, 16].

The choice of cell culture medium also affected the binding of pre-F-specific antibodies to infected cells, with lower normalised binding signals in cells cultured in media B and C than in medium A. Because the relative F/N ratios in the virus stocks did not differ significantly between the virus preparations, these differences were not accompanied by detectable differences in relative F protein signal in the virus-stock Western blot analysis. However, this analysis did not distinguish between pre-F and post-F conformations, and the infected-cell staining assay does not establish the underlying basis of the observed binding differences. Importantly, the IC_50_ values determined for viruses produced in media B and C were not significantly different, indicating that the observed differences in infected-cell antibody-binding signals were not accompanied by a statistically significant difference in neutralising activity under the conditions tested. IC_50_ values could not be reliably determined for virus produced in medium A because its attenuated spread kinetics in the IncuCyte-based readout did not permit reliable dose-response analysis.

Elucidating the precise differences in medium-dependent glycosylation patterns of the G protein would require site-resolved glycoproteomic analysis. Furthermore, it would be of interest to also investigate differences in glycosylation of the F protein and a potential effect on the distribution of pre-F and post-F conformations, which also may have contributed to differential antibody binding. In addition, we have used transformed cell lines for our studies. Whether medium-dependent differences in glycosylation and viral output of similar magnitude occur in primary airway epithelial, ALI or airway organoid culture systems remains to be determined [5, 17]. Of note, such cultures are maintained in differentiation media that differ substantially in composition from the media used for continuous cell lines, so the higher apparent molecular weight of G reported in primary cultures may reflect the culture medium in addition to cell identity [5]. The observed medium-dependent differences may be restricted to the specific experimental conditions used here, including the use of the RSV A-0594-EGFP strain, and assay readouts, and may not extend to other viruses and experimental systems. The exact composition of the respective media is proprietary information and was not made available to us, which precluded identification of component(s) responsible for the observed differences. In addition, medium B is a reduced-serum rather than a serum-free formulation, so differences involving medium B may also reflect its serum content and lower nutrient availability. Of note, this study was not intended to compare the overall quality of the respective media. Rather, it identified the culture medium as an experimental variable that should be considered specifically when producing and characterizing RSV stock preparations.

In conclusion, this work identified the choice of cell culture medium as an important variable that should be considered for the production of RSV preparations used in studies aiming at the investigation of biological and immunological properties of RSV. This also implies the use of clinically relevant experimental systems and 3D cell culture models.

## Materials and methods

### Cell lines and culture media

Cell lines were STR-authenticated and routinely tested negative for mycoplasma (MycoStrip, InvivoGen). Three commercial media were used: Medium A (EX-CELL^®^ 293 Serum-Free Medium, Sigma-Aldrich), Medium B (Opti-MEM™ I Reduced Serum Medium, Gibco), and Medium C (VaccineXpress™, Cytiva). Opti-MEM (medium B) served as the standard reference medium throughout. Media A and C are serum-free formulations, whereas medium B is a reduced-serum medium. Medium B was used as the reference because it is commonly used for RSV propagation. Nine commercial cell culture media were initially screened for their effect on the apparent molecular weight of recombinant sG produced in HEK293T cells (data not shown). The media screened were EX-CELL^®^ 293 (Sigma-Aldrich), Opti-MEM™ I (Gibco), Panserin 293 A (PAN-Biotech), HyClone™ VaccineXpress, HyClone™ PEAK, HyClone™ PRIME and HyClone™ SFM4HEK293 (Cytiva), SMM 293-TII (Sino Biological) and Pro293a-CDM (Lonza). The three media were selected for detailed characterization to represent the extremes and midpoint of this range.

### G protein purification and characterization

RSV A soluble G (sG) protein with a C-terminal 6×His tag [18] was expressed in transiently transfected HEK293T and glycosylation-deficient HEK293T GALE/GALK1 knockout cells (EMI017, Kerafast) maintained in media A, B, or C. The sG protein is the naturally secreted form of G, which initiates at the second in-frame methionine (Met48) and lacks the cytoplasmic tail and transmembrane domain while retaining the 16-amino-acid leader sequence directing its secretion. Clarified supernatants were purified by immobilized metal affinity chromatography (HisTrap™ excel, Cytiva), dialyzed against PBS with protease/phosphatase inhibitors (Abcam), and quantified by Bradford assay (BioRad). To assess G protein size in the context of authentic infection, A549 cells were infected with RSV A-0594-EGFP at MOI 1 in media A, B, or C and lysed at 24 hpi using M-PER lysis buffer. Lysates and purified protein were analyzed by Western blot using an anti-G antibody (Merck; clone 131-2G).

### Virus production and characterization

A549 cells were infected with RSV A-0594-EGFP (ON1 genotype; [19]) at MOI 0.01 and maintained in media A, B, or C for 5 days. Harvested virus stocks were stabilized with 40% glycerol/FBS and stored at − 150 °C. Viral titers were determined by TCID_50_ on HEp-2 cells. Virus stocks were clarified from cell debris by low-speed centrifugation and were not further purified or concentrated. For RT-qPCR, RNA was extracted from 100 µL of each stock (QIAamp Viral RNA Mini Kit, Qiagen). Amplification of the NS1/NS2 junction was performed using the Luna Universal One-Step RT-qPCR Kit (NEB; 55 °C annealing, 40 cycles) and specific primers (RSVA NS1/2 fwd: CACTGCTCTCAACCTAATGGC; RSVA NS1/2 rev: TGATCATCAGTCTTTGTGGTGTG) to determine Cq values. To assess relative glycoprotein signals in virus stocks, virus-stock samples were adjusted to equal total protein concentrations by Bradford assay, lysed and analysed by Western blot using a polyclonal RSV antibody able to detect G, F and N protein (Abcam, ab1128). G, F and N protein band intensities were quantified by densitometry using Image Lab software (Bio-Rad) from three independent blots to calculate relative G/N and F/N ratios.

### Infection and neutralization assays

For infectivity assays, A549 cells were infected at MOI 1 with virus stocks grown in Media A, B, or C. To assess whether differences in virus spread persisted after replacement of the production medium, A549 cells were infected with virus stocks grown in media A, B or C. The inoculum was removed after 2 h and replaced with standard medium B in all conditions. For neutralisation assays, HEp-2 cells (1.5 × 10³ cells/well) were incubated with serial dilutions of palivizumab, clesrovimab or nirsevimab (MedChemExpress) pre-mixed with RSV stocks at an MOI of 0.1 (1.5 × 10³ TCID_50_) for 30 min at 37 °C. Integrated GFP fluorescence (GCU × µm²/image) was quantified over 5 days using an IncuCyte S3 system (Sartorius). IC_50_ values were calculated by four-parameter logistic nonlinear regression.

### Antibody labeling

HEK293T cells infected with RSV-A-EGFP (MOI 0.1) were cultured in media A, B, or C. At 24 hpi, cells were fixed (4% PFA) and blocked (3% BSA). Cells were stained (1:300, 1 h) with therapeutic antibodies targeting RSV F labeled with DyLight^®^ 594 and a postF-specific control antibody (MAB8599, Merck) labeled with DyLight^®^ 350 using Lightning-Link^®^ Fast Conjugation Kits (Abcam). Fluorescence intensity was quantified using a Tecan microplate reader (normalized to eGFP) and imaged on a Leica DM8 fluorescence microscope.

### Statistical analysis

Analyses were performed in GraphPad Prism 10.0.3. Data are means ± SD. Significance thresholds: ns = *p* > 0.05; * = *p* ≤ 0.05; ** = *p* ≤ 0.01; *** = *p* ≤ 0.001; **** = *p* ≤ 0.0001. Specific tests are detailed in figure legends.

## Supplementary Information

Supplementary Material 1

## Data Availability

No datasets were generated or analysed during the current study.
