# Supplementary material for: Cell culture medium influences RSV G protein glycosylation and recognition by monoclonal antibodies
Source: Virol J. 2026 Sep 29;23:226. doi: 10.1186/s12985-026-03318-8 (PMC13621597; doi:10.1186/s12985-026-03318-8)
Supplement: Supplementary file 1 [file 12985_2026_3318_MOESM1_ESM.docx]

# Uncropped Gels and Blots image(s)

Engling et al., "Cell culture medium influences RSV G protein glycosylation and recognition by monoclonal antibodies". Blots are listed in the order of the Figure 1 panels in which they appear, and each is identified by panel, row and culture medium.

### Figure 1a, top row - purified soluble G protein produced in HEK293T cells (anti-G, clone 131-2G)

**Blot 1 - Figure 1a, top row (soluble G, HEK293T): medium A (EX-CELL® 293, Sigma-Aldrich). Anti-G, clone 131-2G.**

**Blot 2 - Figure 1a, top row (soluble G, HEK293T): medium B (Opti-MEM™ I, Gibco). Anti-G, clone 131-2G.**

**Blot 3 - Figure 1a, top row (soluble G, HEK293T): medium C (VaccineXpress™, Cytiva). Anti-G, clone 131-2G.**

### Figure 1a, middle row - purified soluble G protein produced in HEK293T GALE/GALK1 knockout cells (anti-G, clone 131-2G)

**Blot 4 - Figure 1a, middle row (soluble G, HEK293T GALE/GALK1 knockout): medium A (EX-CELL® 293, Sigma-Aldrich). Anti-G, clone 131-2G.**

**Blot 5 - Figure 1a, middle row (soluble G, HEK293T GALE/GALK1 knockout): medium B (Opti-MEM™ I, Gibco). Anti-G, clone 131-2G.**

**Blot 6 - Figure 1a, middle row (soluble G, HEK293T GALE/GALK1 knockout): medium C (VaccineXpress™, Cytiva). Anti-G, clone 131-2G.**

### Figure 1a, bottom row; whole-cell lysates of RSV A-0594-EGFP-infected A549 cells, 24 hpi (anti-G, clone 131-2G)

**Blot 7 - Figure 1a, bottom row (RSV-infected A549 whole-cell lysate, 24 hpi): medium A (EX-CELL® 293, Sigma-Aldrich). Anti-G, clone 131-2G. Sample in lane 3.**

**Blot 8 - Figure 1a, bottom row (RSV-infected A549 whole-cell lysate, 24 hpi): medium B (Opti-MEM™ I, Gibco). Anti-G, clone 131-2G. Sample in lane 3.**

**Blot 9 - Figure 1a, bottom row (RSV-infected A549 whole-cell lysate, 24 hpi): medium C (VaccineXpress™, Cytiva). Anti-G, clone 131-2G.**

### Figures 1d and 1e - blots used for G/N and F/N densitometry (polyclonal anti-RSV, ab1128; detects G, F and N)

**Blot 10 - Figures 1d and 1e, independent replicate 1 of 3. Lanes, left to right: medium A, medium B, medium C. Polyclonal anti-RSV (ab1128), detecting G, F and N.**

**Blot 11 - Figures 1d and 1e, independent replicate 2 of 3. Lanes, left to right: medium A, medium B, medium C. Polyclonal anti-RSV (ab1128), detecting G, F and N.**

**Blot 12 - Figures 1d and 1e, independent replicate 3 of 3. Lanes, left to right: medium A, medium B, medium C. Polyclonal anti-RSV (ab1128), detecting G, F and N.**
